# Combination of agomelatine and bupropion for treatment-resistant depression: results from a chart review study including a matched control group

**DOI:** 10.1002/brb3.318

**Published:** 2015-02-19

**Authors:** Kurt-Wolfram Sühs, Christoph Correll, Christian K Eberlein, Refik Pul, Helge Frieling, Stefan Bleich, Kai G Kahl

**Affiliations:** 1 Department of Neurology, Hannover Medical SchoolCarl-Neuberg-Str. 1, Hannover, 30625, Germany; 2 Department of Psychiatry, The Zucker Hillside HospitalGlen Oaks, New York; 3 Department of Psychiatry and Molecular Medicine, Hofstra North Shore Long Island Jewish School of MedicineHempstead, New York; 4 Department of Psychiatry and Behavioral Sciences, Albert Einstein College of MedicineBronx, New York; 5 Department of Psychiatry, Social Psychiatry and Psychotherapy, Hannover Medical SchoolCarl-Neuberg-Str. 1, Hannover, 30625, Germany

**Keywords:** Agomelatine, bupropion, treatment-resistant depression

## Abstract

**Introduction:**

Although a growing selection of antidepressants is available, a significant number of patients do not reach clinical remission, despite multiple trials. Data concerning the efficacy and safety of combination therapies with newer antidepressants are limited.

**Methods:**

Fifteen inpatients with treatment-resistant depression (TRD), defined as Beck Depression Inventory-2 (BDI-2) scores >14 despite treatment with adequate doses of ≥1 antidepressant classes for ≥6 weeks, were treated with agomelatine plus bupropion for ≥6 weeks, and compared to 15 patients on antidepressant monotherapy with TRD matched on age, sex, and TRD stage based on retrospective chart review. The primary outcome was change in BDI-2 scores. Secondary outcomes included treatment response (BDI-2 score decrease by ≥50%), remission (BDI-2 score <13), routinely measured cardiometabolic parameters and adverse effects.

**Results:**

After a mean of 6 ± 1 weeks, BDI-2 scores decreased by 20.3 ± 5.6 points in the combination group compared to 12.5 ± 15.1 points in the monotherapy group (*P *=* *0.073; Cohen's *d* = 0.7). Altogether, 73.3% in the combination group responded compared to 53.3% on monotherapy (*P* = 0.27). About 60.0% on combination therapy reached remission compared to 40% on monotherapy (*P* = 0.28), a difference equivalent to a number-needed-to-treat = 4. Treatment response was independent of the degree of TRD (*P* = 0.27). Bupropion-agomelatine cotreatment was well tolerated and laboratory adverse effect parameters were not altered.

**Conclusion:**

Despite the small sample and uncontrolled study design, the good remission rate in TRD patients receiving agomelatine plus bupropion, particularly in comparison to the monotherapy group, indicates that this combination treatment should be explored further as a potentially promising strategy for patients with TRD.

## Introduction

Major depression is a widespread psychiatric illness with a severe impact on patients’ everyday life. However, many patients with major depressive disorder fail to achieve remission with standard antidepressant therapy. The STAR*D trial showed that half of all patients will respond to an initial trial of an antidepressant, with only a third reaching clinical remission (Rush 2011). Moreover, up to a third of patients may not reach remission despite multiple drug trials (Rush 2011). The underlying causes of this insufficient outcome remain unclear. However, it is known that patients in remission are less likely to relapse (Rush et al. 2006). Current options for the management of treatment-resistant depression (TRD) comprise dose optimization, switching within or between drug classes, augmenting with other drugs, and combination strategies (Philip et al. 2010). Among these strategies, combinations of mirtazapine or mianserine with other first-line antidepressants may prove useful (Connolly and Thase 2011) and are recommended by the German National Disease Management Guideline for unipolar depression in nonresponders (Harter et al. 2010). However, the results of studies investigating the effectiveness of antidepressant cotreatments have been mixed (Blier et al. 2010; Rush et al. 2011; Chernoloz et al. 2012; Stewart et al. 2014) and antidepressant cotreatment may be limited by adverse side effects (van der Klauw et al. 1998; Serretti and Mandelli 2010), and can complicate adherence.

Bupropion is a norepinephrine and dopamine reuptake inhibitor. Although data from placebo controlled trials are lacking that support bupropion's use in antidepressant combination therapy, its comparably low side effect profile make bupropion a favorable augmentation strategy, and smaller studies have implicated its beneficial use in TRD (Maron et al. 2009). In clinical practice, combining bupropion with other antidepressants has become popular, justifying the need for further studies to support or refute its usefulness.

Agomelatine, a 5-HT2C receptor antagonist and agonist on the melatonergic MT1 and MT receptors, has shown antidepressant effects (Loo et al. 2002; Kennedy and Emsley 2006; Olie and Kasper 2007; Kennedy et al. 2008; Goodwin et al. 2009; Guaiana et al. 2013; Taylor et al. 2014), but there are very limited data on its effect in treatment-resistant depression. Agomelatine seemed to be well tolerated in these trials. Unlike bupropion and many other antidepressants, it has no effect on monoamine reuptake, promoting its use in combination treatment with antidepressants that almost all exert their efficacy via modulation of monoamine transmission.

Based on these theoretical and practical considerations, we undertook an open trial of combined use of agomelatine and bupropion in inpatients not responding to first-line antidepressants, comparing outcomes to a matched control group of patients treated naturalistically with antidepressant monotherapy.

## Methods

Reviewing patient charts from February 2010 to February 2011 revealed that 15 inpatients with TRD were treated at the Department of Psychiatry, Social Psychiatry, and Psychiatry at the Hannover Medical School with a combination of agomelatine and bupropion after discontinuation of former antidepressant treatment. These patients were compared to a control group of 15 TRD patients treated with antidepressant monotherapy matched on age, sex, and TRD stage. The study was performed in compliance with the relevant laws, institutional guidelines, and ethic committees. In both groups, data were retrospectively assessed via chart review. TRD was defined according to the five-stage model by Thase and Rush (1997), that is, patients with a BDI-2 score >14 despite treatment with adequate dose of ≥1 different antidepressant classes for ≥6 weeks.

The intensity of depressive symptoms was evaluated weekly using Beck's Depression Inventory-2 (BDI-2) (Beck et al. 1996), which was administered as part of usual clinical care prior to initiation of combination treatment and at discharge. Metabolic and other laboratory parameters were routinely assessed in all patients at the beginning, after 3–4 weeks and at discharge. A linked enzymatic test with measurement of an optic indicator reaction (NADH) was used for assessing laboratory parameters. Reference values for adults (reaction temperature 37°) are: ALT (GPT) male below 45 U/L, female below 34 U/L; AST male below 35 U/L, female below 31 U/L; Glucose (S) 3.9–5.5 mmol/L; HDL male 0.7–1.8 mmol/L, female 0.8–2.0 mmol/L; Triglycerides male 0.47–4.15 mmol/L; 0.40–2.92 mmol/L. Adverse effects were checked and recorded daily as part of usual clinical visits based on patient account.

BDI-2 sum scores were analyzed within group with paired *t*-test and across the two treatment groups with repeated measurements ANOVA with degree of TRD and baseline BDI2-score as a covariate. Effect size was assessed using Cohen's *d* Response was defined as a decrease of BDI-2 sum score reduction of ≥50%, and remission as a BDI-2 sum score <13. Response and remission were compared across groups using regression analyses, adjusting results for degree of TRD, and baseline BDI-2 scores. As an effect size measure for this small pilot study, we calculated the number-needed-to-treat (1/difference in frequency of response and remission across the two groups) even if results were not significant.

## Results

### Baseline and treatment characteristics

Between February 2010 and February 2011, 15 depressed inpatients with TRD were identified and treated with the agomelatine/bupropion combination. These patients were matched to 15 patients treated naturalistically with antidepressant monotherapy during their hospitalization in the same period. The mean age was 46.3 ± 7.4 years in the combination group, compared to 47.4 ± 5.1 in the control group (Table 1). The mean body mass index was 28.1 ± 4.9 and 26.8 ± 5.2. Baseline BDI-2 scores in the combination and monotherapy groups were 32.4 ± 7.0 and 31.9 ± 12, respectively. According to the Thase and Rush (1997) classification, the mean TRD stage in the combination group was 1.40 ± 0.28 compared to 1.33 ± 0.98 in the control group.

**Table 1:** Change in BDI-2 scores with combination treatment with agomelatine plus bupropion compared to treatment with antidepressant monotherapy.

Baseline characteristics	Agomelatine + Bupropion *N* = 15	Antidepressant Monotherapy *N* = 15
Age (year)	46.3 ± 7.4	47.4 ± 5.1
Female sex (*N*/%)	7 (47%)	6 (40%)
Height (meters ± SD)	1.77 ± 0.14	1.75 ± 0.06
Weight (kg ± SD)	89.6 ± 25.2	82.2 ± 18.3
BMI (kg/m^2^ ± SD)	28.1 ± 4.9	26.8 ± 5.2
Mean TRD (range)	1.40 ± 0.28 (1–3)	1.33 ± 0.98 (1–3)
Antidepressants (*N*; mg/day)		
Agomelatine	15/15; 47.9 ± 7.2	
Bupropion	15/15; 325 ± 86.6	
Citalopram		7/15; 34.3 ± 9.8
Venlafaxine		3/15; 350 ± 86.6
Duloxetine		2/15; 105 ± 21.1
Mirtazapine		2/15; 45.0 ± 0
Sertraline		1/15; 150 ± 0
Benzodiazepine (lorazepam)	3/15; 1.0 ± 0.44	3/15; 1.0 ± 0.44
Follow-up duration (days)	40.9 ± 7.2	43.8 ± 7.3
Outcomes	Combination treatment	Monotherapy
Baseline BDI-2 score (±SD)	32.4 ± 7.0	31.9 ± 12.1
Endpoint BDI-2 score (±SD)	12.1 ± 8.7	19.4 ± 17.5
Change in BDI-2 score (±SD)	−20.3 ± 5.6	−12.5 ± 15.1
Response (*N*/%)	11/15 (73.3%)	8/15 (53.3%)
Remission (*N*/%)	9/15 (60.0%)	6/15 (40.0%)
All-cause discontinuation (*N*/%)	0/15 (0%)	0/15 (0%)
Discontinuation due to inefficacy (*N*/%)	0/15 (0%)	0/15 (0%)
Discontinuation due to intolerability (*N*/%)	0/15 (0%)	0/15 (0%)

BDI-2, Beck Depression Inventory-2; BMI, body mass index; SD, standard deviation; TRD, treatment-resistant depression.

Before admission to the hospital, all patients had been treated with selective serotonin reuptake inhibitors for ≥6 weeks. Six of them then received either selective serotonin or norepinephrine reuptake inhibitors (4/15) or mirtazapine (2/15) for another 6–8 weeks. One of these was subsequently treated with amitriptyline. Combination treatment was started with agomelatine (25 mg/day) and bupropion (150 mg/day) and titrated according to clinical impression. Patients were treated with this combination therapy for 6 ± 1 weeks (40.9 ± 7.2 days in the combination group, 43.8 ± 7.3 days in the monotherapy group). Mean antidepressant doses in the combination group at discharge were 47.9 ± 7.2 mg/day for agomelatine, and 325 ± 86.6 mg/day for bupropion. Treatment in the monotherapy group consisted of: citalopram (*N* = 7; mean daily dose 34.3 ± 9.8 mg), venlafaxine (*N* = 3; mean daily dose 350 ± 86.6 mg), duloxetine (*N* = 2; mean daily dose 105 ± 21.1 mg), mirtazapine (*N* = 2; mean daily dose 45 ± 0 mg), and sertraline (*N* = 1; daily dose 150 mg). Three patients in the combination group and three patients in the comparison group received additional lorazepam (0.5–1.5 mg/day) (see Table 1).

### Efficacy

After 6 ± 1 weeks, the mean BDI-2 score improved from a baseline score of 32.4 ± 7.0 to 12.1 ± 8.7 (*P* ≤ 0.001) in the combination group, and from 31.9 ± 12.1 to 19.4 ± 17.5 (*P* = 0.006) in the monotherapy comparison group (Table 1). The antidepressant combination group improved at a trend level more compared to the monotherapy group (−20.3 ± 5.6 vs. −12.5 ± 15.1, *P* = 0.073). Improvement of depressive symptoms was more effective in the agomelatine-bupropion combination treatment group compared to antidepressant monotherapy (Cohen's *d* = 0.7).

Moreover, 11 (73.3%) of the 15 patients in the combination group responded, compared to eight (53.3%) in the control group (*P* = 0.71), a difference translating into a number-needed-to-treat of 5. Treatment response was independent of the degree of TRD (*P* = 0.27). Nine patients on combination therapy (60.0%) reached remission compared to six (40.0%) in the control group (*P* = 0.28) (Table 1), a difference translating into a number-needed-to-treat of 4. Remission was also independent of the degree of TRD (*P* = 0.33).

### Adverse effects

Combination treatment and monotherapy were generally accepted well. None of the patients discontinued treatment for unwanted side effects in both groups. However, chart analysis revealed that four patients in the combination group reported headache for approximately 1 week, and three patients reported increased motor activity or agitation. Routinely assessed factors of the metabolic syndrome revealed no significant changes from baseline in both treatment groups, in particular no changes in liver enzymes (Table 2).

**Table 2:** Factors determining the metabolic syndrome and liver enzymes in patients treated with antidepressant combination or monotherapy.

	Combination group	Monotherapy group	*P*
Systolic BP (mmHg)			
T0	137.6 ± 18.4	135 ± 22.8	n.s.
T1	124.6 ± 17.4	129.3 ± 13.2	n.s.
Diastolic BP (mmHg)			
T0	85.7 ± 12.8	86.3 ± 7.9	n.s.
T1	80.0 ± 11.7	80.7 ± 8.2	n.s.
Waist circumference (cm)			
T0	100.6 ± 17.4	98.5 ± 17.0	n.s.
T1	97.3 ± 17.0	95.7 ± 15.2	n.s.
Fasting glucose (mg/dL)			
T0	5.7 ± 1.3	5.8 ± 1.0	n.s.
T1	5.4 ± 1.2	5.8 ± 1.9	n.s.
Triglycerides (mg/dL)			
T0	1.80 ± 1.06	1.77 ± 0.90	n.s.
T1	1.46 ± 0.55	1.76 ± 1.54	n.s.
HDL-cholesterol			
T0	1.48 ± 0.46	1.48 ± 0.36	n.s.
T1	1.46 ± 0.29	1.33 ± 0.27	n.s.
GOT (U/L)			
T0	21.7 ± 4.4	22.4 ± 5.5	n.s.
T1	23.1 ± 3.9	24.4 ± 4.0	n.s.
GPT (U/L)			
T0	19.6 ± 5.7	20.5 ± 5.2	n.s.
T1	22.3 ± 4.7	20.5 ± 5.7	n.s.

BP, blood pressure; GOT, glutamate oxaloacetate transaminase; GPT, glutamic-pyruvic transaminase; HDL, high-density lipoprotein.

## Discussion

The question which treatments to choose when patients have failed to respond to first-line antidepressant medications is an important one. An effective choice can improve outcomes, while persistent depression may lead to a more chronic and deteriorating course (Tranter et al. 2002). Several options exist, including antidepressant switching, augmentation with antidepressants with different mechanisms, augmentation with non-antidepressant psychotropic, such as second-generation antipsychotics or lithium, nonpsychotropic medications (thyroid hormone), or nonpharmacologic treatments (psychotherapy, transcranial magnetic stimulation, electroconvulsive therapy) (Fleurence et al. 2009; Papakostas 2009; Spielmans et al. 2013). However, guidance as to which options are most efficacious or have the best risk-benefit profile remains limited. In particular, the use of antidepressant combinations to address TRD is understudied, although up to one-third of patients will not reach clinical remission after multiple drug trials and although antidepressant combinations may have a favorable adverse effect profiles than augmentation with antipsychotics or lithium (Rush et al. 2011; Rocha et al. 2012).

We chose to explore the combination treatment with agomelatine plus bupropion for patients with TRD because of different and potentially complementary modes of action and favorable side effect profiles. Agomelatine is an agonist at melatonergic MT_1_ and MT_2_ receptors and an antagonist at 5HT_2c_ receptors, and bupropion acts via inhibition of dopamine and norepinephrine reuptake. Both antidepressants are reported to be associated with only moderate side effects (Moreira 2011; Guaiana et al. 2013). In particular, sedation, weight gain, and sexual dysfunction are rare. In this chart review, we have shown that the combination treatment with agomelatine plus bupropion in TRD led to significant symptom relief in 73% of patients and to remission in 60% of patients treated in an inpatient practice setting. Both patients on combination treatment and on monotherapy improved regarding depression ratings. Compared to a group of TRD patients treated naturalistically with antidepressant monotherapy, there was a 7.8 point greater decrease in the BDI-2 score in the combination treatment group, which was showed a trend level superiority favoring the combination group. The difference in antidepressant efficacy has a moderate effect size according to Cohen's *d* (Cohen's *d* = 0.7), which reflects a strong advantage of the combination therapy as similar effect sizes in antidepressant treatment studies are generally achieved when comparing an active treatment with placebo. Moreover, although not statistically significant in this small pilot sample, response was 20% higher, translating into an NNT of 5, and remission was 26.7% higher in the combination treatment group, a difference translating into an NNT of 4. Finally, the combination treatment was well tolerated and unexpected side effects did not occur.

Several limitations of this study are relevant. These include the small sample size of the treatment and the control group, lack of randomization, and short duration. Especially the sample size and short duration have to be taken in account with respect to the side effects in this study. Agomelatine and bupropion have been described to be associated with greater risks of hepatotoxicity and even so liver damage often occurs early during treatment it can be delayed several month (Voican et al. 2014). Therefore, late onset hepatotoxicity might have been missed due to the observation time of 6 weeks in this study. However, despite these obvious drawbacks, our findings imply that antidepressant combination therapy with agomelatine and bupropion may be a valuable treatment option in TRD.

While clearly preliminary, this study suggests that enhancing melatonergic plus dopaminergic neurotransmission and regulating sleep might, at least in SSRI nonresponders, be a promising tool for treating TRD that needs to be explored further. Our finding of potential advantages for achieving remission is particularly important, given data from the Star*D study showing that remission rather than response is associated with an improved long-term prognosis (Rush et al. 2006). To our knowledge this is so far the only study examining the combination treatment of bupropion and agomelatine in TRD. Therefore, future randomized controlled trials are crucial to examine and extend our results and to answer the question of efficacy and safety and of adherence to this combination treatment of bupropion and agomelatine in TRD.

## Conflict of Interest

None declared.
